# External Barrier and Internal Attack: Synergistic Effect of Microcapsule Fire Extinguishing Agent and Fine Water Mist on Suppressing Lithium-Ion Battery Fire

**DOI:** 10.3390/ma18133082

**Published:** 2025-06-29

**Authors:** Xiangjian Wang, Zhanwen He, Jianjun Gao, Yibo Guo, Haijun Zhang, Mingchao Wang

**Affiliations:** 1 Key Laboratory of Civil Aviation Thermal Hazards Prevention and Emergency Response, Civil Aviation University of China, Tianjin 300300, China; 2 School of Safety Science and Engineering, Civil Aviation University of China, Tianjin 300300, China; 3 College of Science, Civil Aviation University of China, Tianjin 300300, China

**Keywords:** lithium-ion battery thermal runaway, jet fire, microcapsule fire extinguishing agent, fine water mist, synergistic effect

## Abstract

When lithium-ion batteries experience thermal runaway, a large amount of heat rapidly accumulates inside, causing the internal pressure to rise sharply. Once the pressure exceeds the battery’s safety valve design capacity, the valve activates and releases flammable gas. If ignited in a high-temperature environment, the escaping gas can cause a jet fire containing high-temperature substances. Effectively controlling the internal temperature of the jet fire, especially rapidly cooling the core area of the flame during the jet process, is important to prevent the spread of lithium-ion battery fires. Therefore, this work proposes a strategy of a synergistic effect using microcapsule fire extinguishing agents and fine water mist to achieve an external barrier and an internal attack. The microcapsule fire extinguishing agents are prepared by using melamine–urea–formaldehyde resin as the shell and 1,1,1,2,2,3,3,4,4-nonafluoro-4-methoxybutane (C_5_H_3_F_9_O) and 1,1,2,2,3,3,4-heptafluorocyclopentane (C_5_H_3_F_7_) as the composite core. During the process of lithium-ion battery thermal runaway, the microcapsule fire extinguishing agents can enter the inner area of the jet fire under the protection of the fine water mist. The microcapsule shell ruptures at 100 °C, releasing the highly effective composite fire suppressant core inside the jet fire. The fine water mist significantly blocks the transfer of thermal radiation, inhibiting the spread of the fire. Compared to the suppression with fine water mist only, the time required to reduce the battery temperature from the peak value to a low temperature is reduced by 66 s and the peak temperature of the high-temperature substances above the battery is reduced by 228.2 °C. The propagation of the thermal runaway is suppressed, and no thermal runaway of other batteries around the faulty unit will occur. This synergistic suppression strategy of fine water mist and microcapsule fire extinguishing agent (FWM@M) effectively reduces the adverse effects of jet fires on the propagation of thermal runaway (TR) of lithium-ion batteries, providing a new solution for efficiently extinguishing lithium-ion battery fires.

## 1. Introduction

Lithium-ion batteries, with a high energy density, long cycle life, and environmental friendliness, have been widely applied in the fields of energy storage systems, new energy vehicles, electric aircraft, and mobile devices [1,2]. However, lithium-ion batteries can experience thermal runaway under abusive conditions such as collision, compression, short circuit, external heating, and overcharging, which may further lead to fire or even explosion incidents [3,4,5,6]. Safety issues have become a significant obstacle to the large-scale application of lithium-ion batteries. To enhance the intrinsic safety of lithium-ion batteries, many researchers have conducted extensive studies [7,8,9,10,11,12,13,14]. Examples include using safer cathode materials, improved separator technologies, and flame-retardant or non-flammable electrolytes. However, these invasive material changes can impact the performance of lithium-ion batteries [15,16,17,18]. Therefore, during abnormal conditions, effective and timely emergency response with external fire extinguishing agents is essential for comprehensive control of battery safety risks.

For powdered fire extinguishing agents, ABC dry powder can quickly extinguish LIB fires, but its cooling capacity is limited, leading to re-ignition after 8 s of extinguishing the visible flame. Additionally, the residual powder after spraying can contaminate the protected area and damage precision instruments [19,20,21,22]. Water-based extinguishing agents can reduce the battery temperature to ambient levels, but a large amount of water can cause short circuits in electrical systems. The battery cells within the pack may experience thermal runaway again after being damaged by short circuits [23,24,25]. For gas extinguishing agents, heptafluoropropane (HFC-227ea, 1,1,1,2,3,3,3-heptafluoropropane, C_3_HF_7_) decomposes at high temperatures, producing fluorinated substances such as -CFO, -CF_3_, and -CF_2_. These substances capture free radicals like H, O, and CH_3_, thereby reducing the rate of the combustion chain reaction [26,27,28]. However, when the thermal release rate of a lithium-ion battery is high, the extinguishing efficiency of HFC-227ea in an open environment is limited [29]. Liquid nitrogen has a superior cooling effect on lithium-ion batteries compared to other gas extinguishing agents, capable of reducing the battery surface temperature to below 0 °C. However, due to its high storage and transportation costs, liquid nitrogen fire extinguishing technology has not yet become widespread [30,31]. Novec 1230 (C_6_F_12_O) initially has a negative inhibitory effect on lithium battery fires, but as the dosage increases, its extinguishing effect gradually becomes positive. In summary, although the current fire extinguishing technologies for lithium battery fires has a certain suppression effect, they also have limitations such as short circuits, high costs, and re-ignition.

The fine water mist technology refers to the technique of spraying liquid under a certain pressure to create droplets with 99% of their diameters (Dv0.99) being less than 1000 μm [32]. With advantages such as non-toxicity, low conductivity, and water saving, fine water mist technology has been widely applied in various fire protection fields. Due to the significantly larger surface area of fine water mist compared to the equivalent volume of conventional water, its heat exchange capability with the jet fire of a lithium-ion battery is greatly enhanced, thereby substantially improving the cooling effect. Wang et al. combined the gas extinguishing agent Novec 1230 (3M, Shanghai, China) with fine water mist to suppress lithium-ion battery fires. The combined method showed good effects in suppressing open flames and cooling batteries. However, this fire-extinguishing approach that involves splitting the agents into two locations requires more storage space and more complex piping systems [21,22,33,34]. Additionally, according to several research findings [24,29,35], high-boiling-point fluorinated gas extinguishing agents can interrupt the chain reactions during lithium battery combustion without causing damage such as short circuits to electrical equipment. 1,1,1,2,2,3,3,4,4-nonafluoro-4-methoxybutane (MNFB) is commonly used as a refrigerant in shell-and-tube heat exchangers [36]. MNFB provides a superior cooling effect through the principle of heat absorption by evaporation. When MNFB is released into the air, its molecules transition from a liquid to a gaseous state, absorbing heat from the surrounding environment and thereby lowering the ambient temperature. 1,1,2,2,3,3,4-Heptafluorocyclopentane (F7A) is environmentally friendly and has low toxicity, making it a promising new halon replacement. Scholars have used density functional theory to obtain the possible reaction pathways for the reactions of F7A with hydroxyl and hydrogen radicals at the theoretical level. These reactions play an important role in chemical inhibition of fire [37].

To improve fire extinguishing efficiency for lithium-ion battery fires, fine water mist can be modified by adding additives. These additives can be categorized into physical surfactants, inorganic salt electrolytes, and composite additives [38,39,40,41,42,43]. Additives can alter the physical and chemical properties of water, such as reducing the surface tension of pure water, thereby further decreasing droplet size and increasing penetrability. Due to the different physical properties of water and gas extinguishing agents, they are difficult to mix together. Faced with this challenge, microencapsulated gas fire extinguishing agents can form a uniform emulsion with water, solving the problem of their mutual insolubility [34,44,45]. The complex thermal runaway propagation paths of the batteries require the extinguishing medium to effectively intervene in the jet fire source and absorb heat. Microencapsulation technology offers an innovative solution that is expected to improve the performance of extinguishing media and enhance the capability in direct intervention and heat absorption. Microencapsulation technology is a method of encapsulating substances in capsules ranging from 1 to 1000 μm in size. Originating in the 1950s, it was initially used for dye encapsulation in carbonless copying paper. Nowadays, this technology is widely applied in various industries, including pharmaceuticals, food, and agriculture. Its primary purposes are to protect and control the release of the encapsulated substances. Microcapsules have a core-shell structure with polymers as the shell and liquid or solid as the core [45]. By adjusting the mechanical properties of the shell, the microcapsules respond to high temperatures by releasing gas extinguishing agents. This helps to suppress the initial thermal runaway of lithium-ion batteries and extinguish fires [46,47,48].

This work explores the innovative application of microencapsulation technology in the field of gas extinguishing agents. The concept of “external barrier and internal attack” refers to the combined action of fine water mist and microcapsule fire extinguishing agents in combating lithium-ion battery fires. Fine water mist serves as the external barrier by effectively blocking thermal radiation transfer, inhibiting fire spread, and reducing oxygen concentration around the fire source. Meanwhile, microcapsule fire extinguishing agents penetrate the outer heat flow and reach the flame root, rupturing to release gas extinguishing agents. These agents trigger chemical reactions that capture radicals and interrupt combustion chain reactions, providing an internal attack effect. Fine water mist and microcapsules provide a dual effect of an external barrier and an internal attack, suppressing combustion and preventing fire spread both physically and chemically. Through this technology, liquid extinguishing agents are converted into solid microcapsule particles that can be precisely released within a specific temperature range of 100 °C to 300 °C. By mixing microencapsulated gas fire extinguishing agents with fine water mist, the gas fire extinguishing agents can be directly released at the root of the flame. This mechanism permits the extinguishing agents to act directly on the fire source, achieving more efficient and targeted fire suppression, thereby significantly enhancing the extinguishing performance.

## 2. Materials Preparation and Characterization Methods

The physicochemical properties of heptafluorocyclopentane (F7A, Tokyo Chemical Industry Co., Ltd., Tokyo, Japan) and 1,1,1,2,2,3,3,4,4-nonafluoro-4-methoxybutane (MNFB, Shanghai Upper Chemical Technology Co., Ltd., Shanghai, China) are shown in Table 1. Potassium perfluorooctanesulfonate (Shanghai Meiriel Biochemical Technology Co., Ltd., Shanghai, China) was used as a surfactant, polyvinyl alcohol (PVA, Bidepharm, Shanghai, China) as a dispersant, and polyvinylpyrrolidone (Aladdin Biochemical Technology Co., Ltd., Shanghai, China) as a polymer surfactant. n-Octanol (Macklin, Shanghai, China) was used as a defoaming agent. Melamine (Anhui Zesheng Technology Co., Ltd., Anqing, China), formaldehyde solution (37 wt% aqueous solution, Shanghai Jizhi Biochemical Technology Co., Ltd., Shanghai, China), and urea (Shanghai Aladdin Biochemical Technology Co., Ltd., Shanghai, China) were the main materials for synthesizing melamine–urea–formaldehyde resin. Anhydrous sodium carbonate (Bidepharm, Shanghai, China) and anhydrous citric acid (AR, Xiya Reagent, Zaozhuang, China) were used to adjust the pH value.

First, 11.65 g of melamine, 11.1 g of urea, 45 g of formaldehyde solution, and 45 g of deionized water were added to a beaker. The pH was adjusted to 9.0 using anhydrous sodium carbonate. The beaker was heated in an oil bath from 25 °C to 70 °C at a rate of 2.5 °C/min while stirring. After maintaining the temperature and stirring for 1 h, the beaker was placed in ice water at 5 °C to quickly lower the temperature below 10 °C. Then, the mixture was diluted with 280 g of distilled water to obtain a 10 wt% polymer prepolymer solution. Next, 15 mL of F7A and 15 mL of MNFB were mixed as the core materials and 150 mL of the 10 wt% polymer prepolymer solution was used as the shell material, along with 0.6 g of potassium perfluorooctanesulfonate, 0.6 g of polyvinyl alcohol, 0.6 g of n-octanol, and 0.08 g of polyvinylpyrrolidone. The pH was adjusted to 5.0 with anhydrous citric acid, then it was stirred at 500 rpm for 6 h, and left to stand for 6 h. Finally, 180 mL of the microcapsule suspension and 420 mL of deionized water were added to a 1 L water-based extinguisher. The extinguisher was pressurized to 1 MPa with nitrogen gas, and a fine water mist nozzle with a droplet diameter of less than 400 μm was used to conduct the lithium battery thermal runaway propagation suppression experiment. Details of the LIBs are shown in Table 2.

The optical microscope (OM) is from HIROX (Tokyo, Japan) with magnification of 35×–2500×, which is used to take optical images of microcapsule fire extinguishing agents after dispersion. The scanning electron microscope (SEM) is a TESCAN MIRA LMS (Tescan, Brno, Czech Republic), with a Pt sputter target and a Schottky field emission electron gun. The resolution is 0.9 nm @ 15 kV (secondary electron image) and 2.0 nm @ 30 kV (backscattered electron image); the magnification ranges from 8× to 100,000×. The transmission electron microscope (TEM) model is a JEM-2100 (JEOL, Tokyo, Japan). This instrument has a magnification range of 2000× to 1,500,000×, and when combined with an energy-dispersive spectrometer (EDS), it can achieve qualitative and quantitative analysis of elemental composition. A German Netzsch STA449 F5 (Netzsch, Selb, Germany) was used for Thermogravimetric Analysis (TG). Under a nitrogen atmosphere, the sample was heated from 30 °C to 500 °C at a rate of 10 °C/min, which allowed for the acquisition of the thermal characteristics of the microcapsule fire extinguishing agents. A Thermo Scientific Nicolet iS20 (Thermo Fisher Scientific Inc., Waltham, MA, USA) was used for Perform Fourier Transform Infrared Spectroscopy (FTIR) analysis. The infrared spectrum of the sample was collected with a resolution of 4 cm^−1^, 32 scans, and a wavelength range of 400 to 4000 cm^−1^. The nanoindenter is a Bruker Hysitron TI980 (Bruker, Billerica, MA, USA), which employs displacement-controlled loading, using displacement as the control signal during the loading process. The test included loading, holding, and unloading stages. The maximum load was 50 μN, the loading rate was 300 μN/minute, and the unloading rate was also 300 μN/minute. The contact angle/surface tension measurement was performed using a German Dataphysics DCAT21 model (DataPhysics Instruments GmbH, Filderstadt, Germany). The microcapsule fire extinguishing agent was compressed into a tablet form, and a droplet of deionized water was applied to the tip of a syringe for contact angle measurement. The Pyrolysis Gas Chromatography-Mass Spectrometry (Py-GCMS) system used is a Shimadzu GCMS-QP-2010 (Shimadzu, Kyoto, Japan). In the experiment, the temperature of the high-temperature furnace was set to 500 °C, and the core material contained within the microcapsule fire extinguishing agent was added to study the thermal degradation mechanism of polymers. The particle size analysis was conducted using an OMEC-LS-609 model (OMEC, Zhuhai, China). The wet analysis method was employed to measure the size of insoluble solid particles in the solution medium, yielding a stable and reliable particle size analysis report.

## 3. Results

### 3.1. Materials Characterization

**Figure 1:**
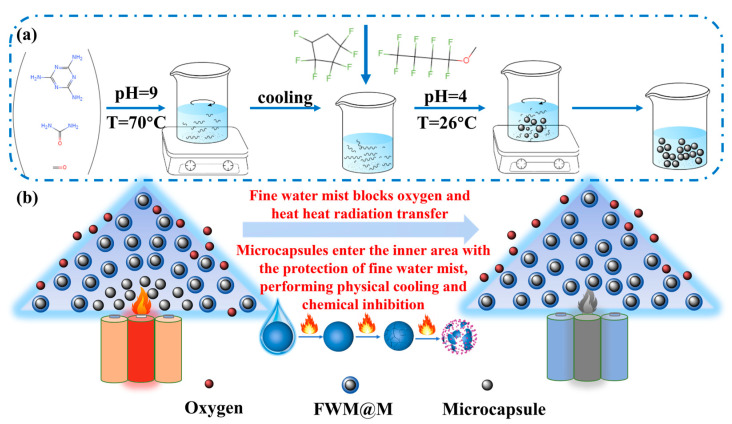
(**a**) The synthesis process of the FWM@M; (**b**) Schematic of the synergistic effect of microcapsule fire extinguishing agents and fine water mist.

The synthesis process of the fine water mist containing microcapsules (FWM@M) is illustrated in Figure 1a. The working principle of the FWM@M is depicted in Figure 1b. The fine water mist effectively blocks the transfer of thermal radiation and inhibits the spread of fire. The fine water mist has excellent coverage capabilities, effectively enveloping the exterior of lithium-ion battery fires. By reducing the oxygen concentration around the combustible material or within the protected area, a fine water mist achieves efficient oxygen depletion, enhancing the smothering effect on the fire source. Protected by fine water mist droplets, the microcapsule fire extinguishing agents can pass through the outer heat flow and reach the root of the flame. The microcapsule fire extinguishing agents rupture in the fire and release the internal gas fire extinguishing agents. This rupture process is crucial as it triggers a series of chemical reactions at high temperatures. The fluorides produced during decomposition, such as -CFO, -CF_3_, and -CF_2_, capture radicals like H, O, and CH_3_, thereby interrupting the chain reactions of combustion. This interruption plays a key role in the chemical suppression of lithium-ion battery fires. The synergistic effect of fine water mist and microcapsule fire extinguishing agents can prevent the spread of jet fire radiation from lithium-ion batteries on the outside, and play a dual role of chemical and cooling effects inside the flame, achieving the effect of external barrier and internal attack.

**Figure 2:**
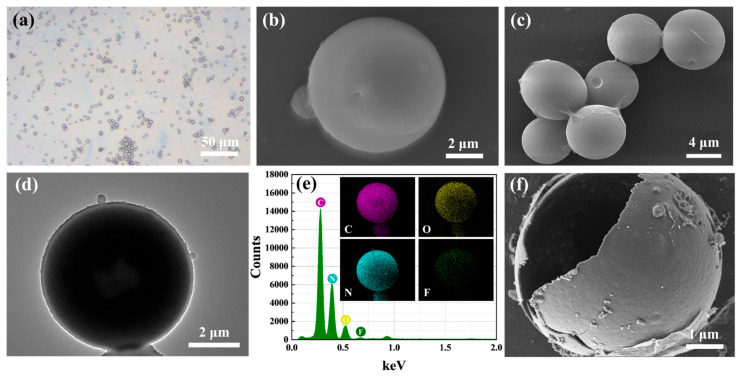
(**a**) Optical image of the microcapsule fire extinguishing agent; (**b**,**c**) SEM images of the microcapsule fire extinguishing agent; (**d**) TEM image of the microcapsule fire extinguishing agent; (**e**) Elemental mapping distribution of C, F, N, and O in the microcapsule fire extinguishing agent; (**f**) SEM image of a microcapsule shell after thermal treatment.

The morphology of the microcapsule fire extinguishing agents was observed with OM, SEM, and TEM. As shown in Figure 2a–c, the images reveal that the microcapsule fire extinguishing agents exhibit a regular spherical shape with a particle size of approximately 5–20 μm. The TEM image reveals a distinct core–shell structure of the microcapsules (Figure 2d), and the mappings show that elements C, N, O and F are evenly distributed (Figure 2e). The residue after extinguishing the fire is shown in Figure 2f. The image shows a microcapsule that ruptured at high temperature, releasing the internal extinguishing agent completely, leaving only the microcapsule shell behind.

**Figure 3:**
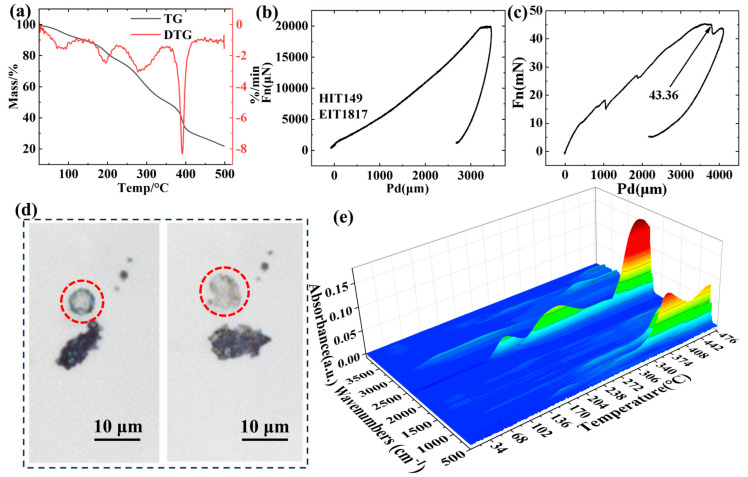
(**a**) TG-DTG curves of the microcapsule fire extinguishing agent; (**b**,**c**) Nanoindentation curves of the microcapsules; (**d**) The in situ imaging nanoindentation before and after the damage of the microcapsule fire extinguishing agent; (**e**) In situ Fourier transform infrared (FTIR) spectrum of the microcapsule fire extinguishing agent.

The TG-DTG curves are shown in Figure 3a. As the temperature increases, the mass of the microcapsules begins to significantly decrease at 100 °C, with a mass loss exceeding 40% between 100 °C and 300 °C. This is due to the extensive vaporization of the internal fire extinguishing agent, and this temperature range is the concentrated release interval for the fire extinguishing agent core material to rupture the polymer shell. The rate of mass decrease reaches another peak at 390 °C, at which point the microcapsule shell undergoes decomposition. The mechanism of extinguishing agent release is as the temperature increases, the liquid core material vaporizes upon reaching the boiling point and the high vapor pressure ruptures the polymer shell to achieve release. The release of the microcapsule fire extinguishing agent core material is not only related to the type of core material but also to the strength and elastic modulus of the shell material. In situ imaging nanoindentation was used to characterize the differences in elastic modulus and hardness. Figure 3b shows the nanoindentation displacement-load curves of the microcapsules, as well as their images from the pressing to the rupture process. It can be concluded from Figure 3b that the hardness of the microcapsule fire extinguishing agent is 149, and the elastic modulus is 1817. As shown in Figure 3c, the load was increased until the microcapsules ruptured, and the pressure at the moment of just breaking was 43.36 μN. The in situ imaging nanoindentation before and after the damage is shown in Figure 3d.

The in situ Fourier infrared spectroscopy of the microcapsule fire extinguishing agents is shown in Figure 3e. The infrared peak near 2400 cm^−1^ is related to the stretching vibrations of the carbon–fluorine (C-F) bonds in MNFB and F7A. In perfluorinated compounds, due to the high electronegativity of fluorine atoms, the vibrational frequency of the C–F bonds tends to be higher [49,50,51,52]. The absorption peak of the core material gas fire extinguishing agent appears at 100 °C, which also indicates that the microcapsule begins to rupture when it reaches 100 °C. As the temperature continues to rise, the release efficiency of the microcapsules reaches its maximum at about 300 °C, and the release amount of the extinguishing agent also reaches its peak. The above test results not only establish the initial temperature for the rupture and release of the microcapsules but also clarify the temperature corresponding to their maximum release amount, providing a theoretical basis for the microcapsules to achieve deep fire extinguishing within a specific temperature range.

### 3.2. High-Temperature Pyrolysis Characteristics of Core Material

**Figure 4:**
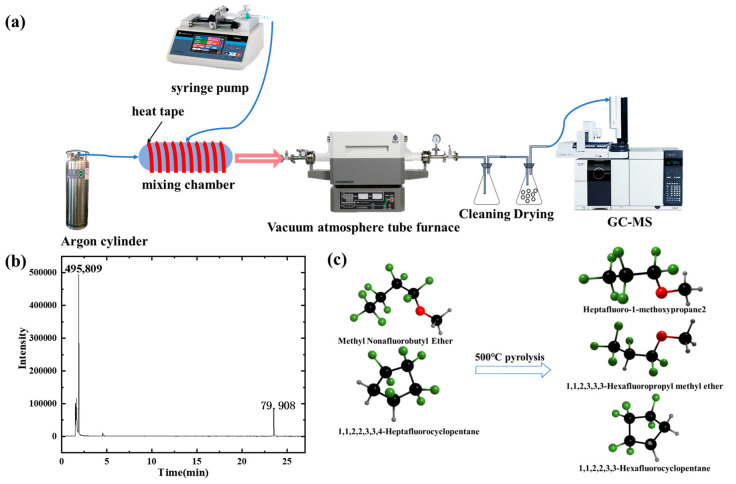
(**a**) High-temperature pyrolysis experimental setup for liquid fire extinguishing agents; (**b**) Mass spectrum of the microcapsule core material pyrolysis at 500 °C; (**c**) Images of the core material before and after pyrolysis at 500 °C.

The high-temperature pyrolysis experimental setup for the core material of fire microcapsule extinguishing agent is shown in Figure 4a. As shown in Figure 4b, the pyrolysis products of the microcapsule core material at 500 °C include 1,1,2,3,3,3-hexafluoropropyl methyl ether, 1,1,1,2,2,3,3-heptafluoro-3-methoxypropane, and 1,1,2,2,3,3-hexafluorocyclopentane. Combustion is essentially a free radical chain reaction, which can be described by a series of chemical equations. In this process, the generation and consumption of free radicals sustain the combustion. Free radicals, such as hydrogen radicals (H∙), are key participants in the combustion reaction. They react with fuel molecules to produce more free radicals, thereby maintaining the chain reaction. The fluorine radicals (F∙) released from the microcapsule core material at high temperatures can react with hydrogen radicals (H∙) to form stable hydrogen fluoride (HF). This reaction effectively consumes hydrogen radicals, interrupting the chain reaction and achieving chemical inhibition. The chemical equation can be represented as follows:

FH → F·+ H·(1)

O_2_ + H·→ O·+ HO (2)

FH → F·+ H_2_ (3)

O·+ H_2_ → HO·+ H· (4)

HO·+ H_2_ → H_2_O + H· (5)

### 3.3. Contact Angle and Particle Size Analysis

**Figure 5:**
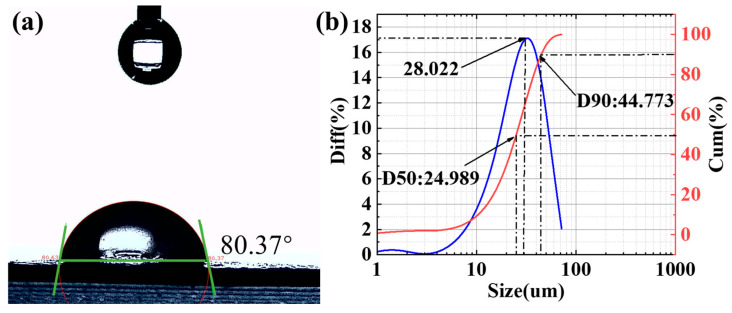
(**a**) Image of droplet contact angle; (**b**) Particle size differential distribution and cumulative distribution curves.

The contact angle is defined as the angle θ formed by the tangent to the gas–liquid interface and the solid–liquid interface at the junction of the gas, liquid, and solid phases. The value of this angle indicates the wettability of the liquid on a solid surface. A contact angle of 0° indicates perfect wetting, where the liquid spreads completely on the solid surface. A contact angle of 180° indicates high hydrophobicity, where the liquid cannot spread and forms a droplet. Contact angles between 0° and 180° indicate partial wetting. This study used video optical contact angle measurement based on sessile drop analysis. The shape of the droplets was captured with a microscope and camera (Figure 5a), and digital image processing and algorithms were used to calculate the contact angles. The contact angle between the microcapsule fire extinguishing agent and water is 80.37°, indicating hydrophilicity. This allows the fine water mist droplets to effectively encapsulate the microcapsules. A contact angle not less than 45° indicates that the polymer shell of the microcapsule is not fully wetted by water, providing good protection for the internal gas extinguishing agent and preventing the core material from failing. Additionally, the surface free energy of the microcapsules was tested, yielding a value of 9.2 mN/m. The surface free energy is the result of both polar and dispersive components. The polar component is 0, representing short-range chemical forces, while the dispersive component is 9.2 mN/m, representing long-range physical forces. This result confirms that the hydrophilicity of the microcapsules is due to physical forces, and that the fine water mist does not chemically react with the microcapsules.

The particle size of the FWM@M emulsion samples was analyzed using a laser particle size analyzer. As shown in Figure 5b, the particle size differential distribution curve provides a clear view of the particle size information for concentrated and dispersed regions. The main particles of the current sample are concentrated around 28.022 μm (median size). The cumulative particle size distribution curve shows that the particle size at the 50% cumulative distribution is 24.989 μm. The particle size at the 90% cumulative distribution for the FWM@M is 44.773 μm.

### 3.4. Lithium-Ion Battery Fire Suppression Experiments

**Figure 6:**
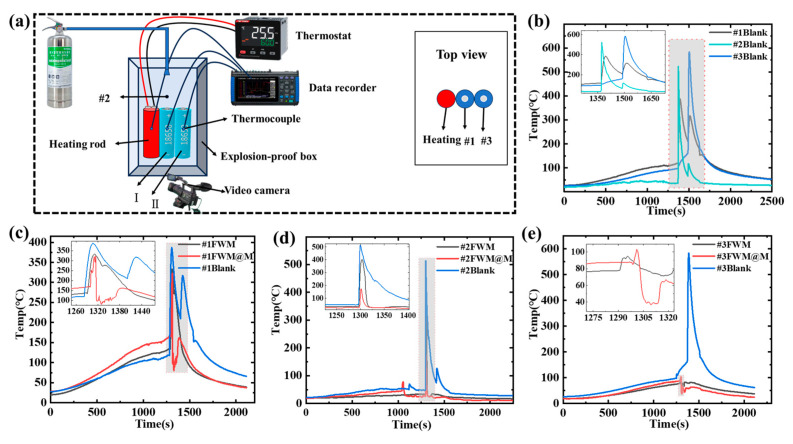
(**a**) Experimental setup for the lithium-ion battery fire suppression test; (**b**) Temperature variation over time measured by three thermocouples under conditions without fire suppressant; (**c**) Temperature comparison measured by Thermocouple #1; (**d**) Temperature comparison measured by Thermocouple #2. (**e**) Temperature comparison measured by Thermocouple #3.

The experimental setup for the lithium-ion battery fire suppression tests is shown in Figure 6a. The experiment was conducted in a closed explosion-proof box at room temperature. A high-power heating rod was used for continuous heating. Two 18,650 NCM lithium-ion batteries were placed side by side next to the heating rod, ensuring close contact to facilitate heat transfer primarily through conduction. The temperatures of the batteries and heating rod were recorded using a temperature data acquisition device. K-type thermocouples (measurement range 0 °C~1300 °C) were fixed to the surface of battery I and II with thermal insulation tape (#1, #3). One additional thermocouple (#2) was positioned 12 cm above the battery I. The target temperature for the heating rod was set at 300 °C, with a heating rate of 20 °C/min. An extinguisher containing FWM@M was placed externally, pressurized to 1 MPa with nitrogen gas and connected to a fine water mist nozzle inside the explosion-proof box via a corrugated tube. Upon thermal runaway of the lithium-ion battery, the extinguisher was activated to release the fire extinguishing agents. For the control comparison, battery fire suppression experiments with and without fine water mist was conducted under the same conditions. It is worth noting that the experimental environment temperature was around a room temperature of 25 °C, with an average humidity of 56%, which is a relatively conventional environmental condition. Due to the characteristics of water-based fire extinguishing agents, this synergistic fire-extinguishing strategy is suitable for use in situations above 0 °C. Battery fires develop rapidly and intensely, and the intervention time for fire-extinguishing measures is relatively short. Variations in temperature and humidity within the normal range have a relatively minor impact on the experimental results.

Figure 6b displays the temperature curves measured by three thermocouples over time under conditions without fire suppressant inhibition. Battery I experienced thermal runaway after 1371 s of heating, with a peak temperature of 387 °C. Additionally, the temperature 12 cm above battery I reached 523.1 °C. After 118 s, battery II underwent thermal runaway, with a more severe temperature change. The peak temperature of battery II was 583.2 °C. Figure 6c shows the temperature changes of battery I monitored by thermocouple #1 under different conditions. With FWM (fine water mist only) suppression, the battery surface temperature dropped from 332.8 °C to 96.4 °C within 76 s. With FWM@M suppression, the temperature sharply decreased from 320.4 °C to 96.4 °C within 10 s, highlighting the significant extinguishing and cooling effect of the FWM@M. The time required to reduce the battery temperature from the peak value to low temperature was reduced by 66 s compared to the suppression of fine water mist only. Figure 6d displays the temperature change curves of thermocouple #2 located 12 cm above battery I under different conditions. With FWM suppression, the highest temperature at this position was 401.2 °C. With FWM@M suppression, the microcapsules entered the inner region of the flame protected by fine water mist. The composite gas fire suppressant within the microcapsules (MNFB and F7A) decomposed at high temperatures and produced fluorine compounds that capture free radicals during combustion, interrupting the chain reaction and significantly reducing the maximum temperature to 173 °C. Compared to the suppression of fine water mist only, the peak temperature of the high-temperature substances above the battery was reduced by 228.2 °C. Figure 6e shows the temperature change of the surface of battery II monitored by thermocouple #3 under different conditions. Compared to the blank control group experiment, no thermal runaway occurred in battery II under both extinguishing agent interventions. With FWM suppression, the battery surface temperature decreased from 93.7 °C to 71.8 °C in 23 s. With FWM@M suppression, the temperature dropped significantly from 103.5 °C to 37.9 °C in just 12 s, which further verified the excellent cooling efficiency of FWM@M.

**Figure 7:**
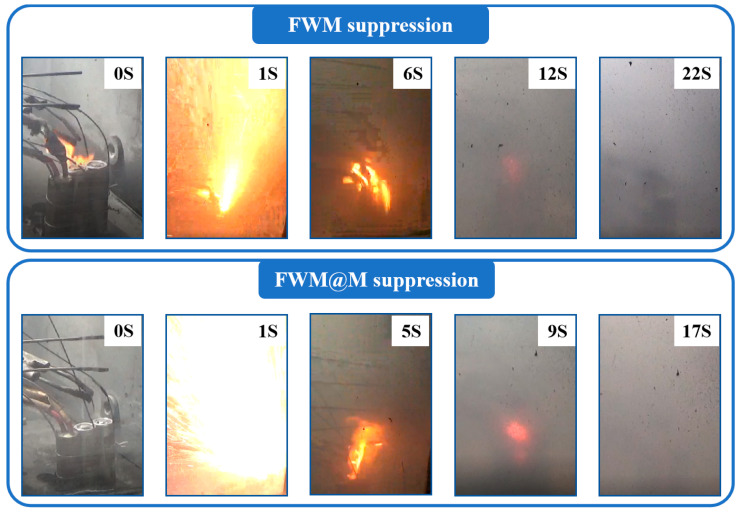
Digital photos of the extinguishing effect of FWM and FWM@M.

Figure 7 shows the digital photos of the extinguishing effect of FWM and FWM@M. Thermal runaway of the battery generates intense jet fires. For the FWM suppression, the flame gradually weakens and is almost completely extinguished by 22 s. For the FWM@M suppression, the flame gradually weakens and is almost completely extinguished by 17 s. Overall, the FWM@M method is more effective in flame suppression. The above results clearly indicate the synergistic effect of microcapsule fire extinguishing agent and fine water mist. FWM@M can effectively extinguish a lithium-ion battery fire, rapidly cool it down, and prevent the thermal runaway propagation, showing the dual effects of external barrier and internal attack.

To explore the application prospects of the fire-extinguishing agent microcapsules prepared in this study, their actual performance was compared with data reported in other literature (Table 3). It can be seen that the microcapsules in this study can achieve better performance at a lower cost compared with those in other literature, demonstrating the excellent fire-extinguishing effect of the product developed in this research.

## 4. Conclusions

To effectively mitigate the adverse effects of jet fires carrying large amounts of high-temperature materials on the thermal runaway and its thermal runaway propagation in lithium-ion batteries, this study proposes a fire-extinguishing strategy that employs the synergistic action of microencapsulated fire extinguishant and fine water mist to achieve both external barrier and internal attack. The microencapsulated fire extinguishant is a novel type of extinguishant, with its shell made of melamine–urea–formaldehyde resin and its core material composed of 1,1,1,1,2,2,3,4,4-nonafluoro-4-methoxybutane (MNFB) and 1,1,2,2,3,3,4-heptafluorocyclopentane (F7A). Fine water mist exhibits remarkably superior performance in blocking the propagation of thermal radiation, thereby suppressing the spread of fire. A systematic investigation was conducted to demonstrate its performance. The main research findings are as follows:

1. The contact angle test indicates that the contact angle between the microcapsule fire extinguishing agent and water is 80.37°, with a surface free energy value of 9.2 mN/m, suggesting the possibility of synergistic action. The in situ infrared test shows that the microcapsule fire extinguishing agent is released at 100 °C. The TG-DTG test reveals that the microcapsule fire extinguishing agent absorbs a large amount of heat within the temperature range of 100 °C to 300 °C. The high-temperature pyrolysis experiment demonstrates that the core material of the fire extinguishing agent can decompose into intermediate components with strong fire extinguishing effects.

2. Under the suppression of FWM (pure water mist), the surface temperature of battery I dropped from 332.8 °C to 96.4 °C within 76 s, and the highest temperature 12 cm above battery I reached 401.2 °C. However, under the suppression of FWM@M, the surface temperature of battery I plummeted sharply from 320.4 °C to 96.4 °C within just 10 s, and the highest temperature 12 cm above battery I only reached 173 °C. The time required for the battery temperature to decrease from its peak to a lower temperature was reduced by 66 s. The highest temperature above the battery was lowered by 228.2 °C. This indicates that FWM@M can effectively extinguish lithium-ion battery fires, rapidly reduce the temperature, and prevent the propagation of thermal runaway.

## Figures and Tables

**Table 1:** Physicochemical properties of core materials.

Material	Molecular Weight [g/mol]	Boiling Point [°C]	Melting Point [°C]	Liquid Density [g/mL]	Vapor Pressure [mmHg]	ODP	GWP	Dielectric Constant [KV]	Heat of Vaporization [KJ/kg]
F7A	196	82.5	21	1.58	11.8	0.0	250	≥110	144
MNFB	250	60	−135	1.52	202	0.0	297	≥25	163

**Table 2:** Details of batteries.

Item	Specification
Battery Model	18,650
Battery Type	lithium-ion battery (NCM)
Weight	48.5 ± 0.5 g
Nominal voltage	3.65 V
State of Charge (SOC)	100%
End Voltage (cut off)	2.75 V
Max Charge Voltage	4.20 V
Battery Capacity	3500 mAh

**Table 3:** Comparison of LIB fire extinguishing agent data.

Agent	Battery Type	Cost	Reusability	Actual Effect	References
Liquid nitrogen	18,650-type	High	No	Liquid nitrogen can effectively cool LIB and suppress TR, without having a significant impact on the cycling performance of LIB.	[30]
C_6_F_12_O + water mist	LiFePO_4_	High	No	The flame of the battery was extinguished by C_6_F_12_O within 1 s, but its cooling effect was poor. When used in combination with water mist, it significantly prolonged the propagation time of TR in the battery, up to 183 s.	[34]
HFC-227ea + water mist	LiFePO_4_	Low	No	HFC-227ea failed to extinguish the flame, but it effectively reduced the fire intensity. When used in combination with fine water mist, it extinguished the flame within 120 s.	[34]
3% F-500 + water mist	18,650-type	Low	No	No re-ignition was observed after the application of 3% F-500 in combination with fine water mist.	[43]
Dry powders	18,650-type	Average	No	The battery fire can be briefly extinguished by the continuous discharge of dry powder for 45 s, but it reignites after 5 s.	[20]
F7A + MNFB + water mist	18,650-type	Low	No	Compared with pure fine water mist, the battery temperature decreases 66 s faster from its peak to a lower temperature, and the peak temperature of the high-temperature material above the battery is reduced by 228.2 °C.	This work

## Data Availability

The original contributions presented in this study are included in the article. Further inquiries can be directed to the corresponding authors.
